# The Microbiome and Gut Endocannabinoid System in the Regulation of Stress Responses and Metabolism

**DOI:** 10.3389/fncel.2022.867267

**Published:** 2022-05-11

**Authors:** Raj Kamal Srivastava, Beat Lutz, Inigo Ruiz de Azua

**Affiliations:** ^1^Department of Zoology, Indira Gandhi National Tribal University, Anuppur, India; ^2^Institute of Physiological Chemistry, University Medical Center of the Johannes Gutenberg University Mainz, Mainz, Germany; ^3^Leibniz Institute for Resilience Research (LIR), Mainz, Germany

**Keywords:** endocannabinoid system, gut microbiota, metabolism and obesity, stress, stress resilience, gut-brain axis

## Abstract

The endocannabinoid system, with its receptors and ligands, is present in the gut epithelium and enteroendocrine cells, and is able to modulate brain functions, both indirectly through circulating gut-derived factors and directly through the vagus nerve, finally acting on the brain’s mechanisms regarding metabolism and behavior. The gut endocannabinoid system also regulates gut motility, permeability, and inflammatory responses. Furthermore, microbiota composition has been shown to influence the activity of the endocannabinoid system. This review examines the interaction between microbiota, intestinal endocannabinoid system, metabolism, and stress responses. We hypothesize that the crosstalk between microbiota and intestinal endocannabinoid system has a prominent role in stress-induced changes in the gut-brain axis affecting metabolic and mental health. Inter-individual differences are commonly observed in stress responses, but mechanisms underlying resilience and vulnerability to stress are far from understood. Both gut microbiota and the endocannabinoid system have been implicated in stress resilience. We also discuss interventions targeting the microbiota and the endocannabinoid system to mitigate metabolic and stress-related disorders.

## Introduction

As stated by Hippocrates “All diseases start in the gut,” now it is time to believe our gut instinct and treat the gut as a master regulator of our physiology. Over the last two decades, a plethora of studies have clearly established the role of the gut microbiota in various metabolic, neurological, and psychiatric disorders. The gut-brain connection is instrumental in molding our physiology, emotional behavior, and stress response (Cathomas et al., 2019). Stress defines a state of mental and physical tension resulting from adverse or demanding circumstances. Therefore, any change and imbalance in the gut microbiome affect our mental and metabolic health.

The endocannabinoid (eCB) system has been shown in rendering physiological and behavioral resilience to stress and now seems to play a part in the regulation of gut-brain axis. Cannabinoids comprise both plant-derived cannabinoids, such as Δ^9^-tetrahydrocannabinol (THC) and cannabidiol (CBD), and the eCBs, which are synthesized endogenously by our body. Cannabis has been in use since several thousands of years for medical, religious, and recreational purposes. The role of the eCB system is largely homeostatic in nature and provides resilience to the body to cope with internal and external adverse conditions. In the central nervous system, the eCB system plays crucial roles in the regulation of stress responses, and allostatic alterations in eCB system activity can lead to behavioral and metabolic disorders (Cani, 2019; Iannotti and Di Marzo, 2021). Although cannabinoids have therapeutic potential in various neurological disorders, however, the use of synthetic and phytocannabinoids targeting the eCB system faces many challenges, owing to multiple receptors and ligands acting through different mechanisms (Di Marzo, 2018). In this review, we summarize the role of the eCB system in gut physiology, stress response and its crosstalk with the gut microbiome.

## The Endocannabinoid System

The preparations from the cannabis plant *Cannabis sativa* (e.g., marijuana and hashish) have been in use for medical purposes for more than two thousand years. They have been used to treat various ailments, including pain, insomnia, anxiety, lack of appetite, and gastrointestinal (GI) discomfort. The eCB system was identified about 30 years ago while investigating the mechanism of action of THC, the psychoactive component of cannabis.

The eCB system participates in a plethora of physiological functions, including stress coping, anxiety, fear responses, social behavior, and energy storage (Silvestri and Di Marzo, 2013; Lutz et al., 2015; Wei et al., 2017; Ruiz de Azua and Lutz, 2019). Notably, the eCB system has been found to be altered in several pathological conditions such as anxiety disorders, post-traumatic stress disorder (PTSD), depression, autism, eating disorders, and irritable bowel syndrome (IBS) along with others. To understand the variety of its functions, the eCB system has been viewed as a homeostatic system instrumental, e.g., in stress recovery (Lutz et al., 2015; Morena et al., 2016), and exostasis (Piazza et al., 2017), the latter being a process that drives energy accumulation and storage. Experimental evidences have also fuelled the stress-induced “hypocannabinergic state” hypothesis, which links a lowered eCB tone to an increase vulnerability to anxiety disorders or PTSD (Bosch-Bouju et al., 2016; Bluett et al., 2017). Despite the crucial role of the eCB system in controlling multiple physiological and pathological processes, only a few compounds acting on this system have been successfully developed for therapeutic purposes (Maldonado et al., 2020). A better understanding of the roles of eCB system in health and disease will help to lead to therapeutic strategies minimizing the risk associated with the use of cannabinoid receptor agonists.

The eCB system is composed of two G-protein coupled receptors (GPCR) (cannabinoid type-1 receptor or CB1R, and cannabinoid type-2 receptor or CB2R), the two main endogenous ligands (called eCBs: *N*-arachidonoyl ethanolamide or anandamide (AEA), and 2-arachidonoyl glycerol or 2-AG) and the enzymes responsible for the synthesis and degradation of eCBs.

The functions of the eCB system are mainly mediated by activating the CB1R and CB2R. While CB2R is highly expressed in the immune system (Munro et al., 1993), but also present in the central nervous system (CNS) both in neurons (Van Sickle et al., 2005; Li and Kim, 2015) and in microglial cells (Maldonado et al., 2020), CB1R is the most abundant GPCR in the brain (Matsuda et al., 1990). Presynaptic CB1R is involved in the canonical eCB-mediated suppression of excitatory or inhibitory synaptic transmission. Besides presynaptic location in neurons, CB1R is also present at post-synaptic terminals (Maroso et al., 2016) and intracellularly in the mitochondria (Benard et al., 2012), as well as in non-neuronal cells such as astrocytes (Navarrete and Araque, 2008; Han et al., 2012). CB1R is also present in peripheral tissues although at much lower levels, including adipose tissues, liver, skeletal muscles, kidney, pancreas and GI tract (Cavuoto et al., 2007; Osei-Hyiaman et al., 2008; Malenczyk et al., 2013; DiPatrizio, 2016; Ruiz de Azua et al., 2017). A widespread expression in various peripheral organs has also been reported for CB2R (Rogers, 2015; Bie et al., 2018).

Endocannabinoids are lipids derived from the membrane phospholipids, and different and redundant pathways have been identified for their synthesis and degradation (Muccioli, 2010). Notably, these pathways also lead to the production of other bioactive lipids, for example, anti-inflammatory palmitoyl ethanolamide (PEA) (Esposito et al., 2014; Petrosino and Di Marzo, 2017), anorexigenic oleoyl ethanolamide (OEA) (Rodriguez de Fonseca et al., 2001) or prostaglandins among others (Kozak et al., 2002). It is noteworthy to consider these other bioactive lipids in pharmacological approaches targeting the pathways involved in synthesis and degradation of endogenous ligands.

Furthermore, eCBs are promiscuous compounds that also interact with other receptors, such as transient receptor potential vanilloid type 1 (TRPV1), peroxisome proliferator-activated receptor-α and -γ (PPARα, PPARγ), and G-protein-coupled receptor 55 (GPR55) (Bouaboula et al., 2005; Ryberg et al., 2007; Di Marzo and De Petrocellis, 2010). There are some inconsistent data whether AEA and 2-AG can activate GPR55, but the recently identified CB1-GPR55 heteromer might explain this controversy (Martinez-Pinilla et al., 2014). The other two members of the *N*-acylethanolamine family, PEA and OEA, can activate PPARα, TRPV1 and GPR119 (GPR119).

The *cannabis* plant contains more than 80 phytocannabinoids. Among them, only THC, the main component responsible for the psychotropic and euphoric effects of the plant, and Δ^9^-tetrahydrocannabivarin have affinity to CB1R and CB2R (Di Marzo and Piscitelli, 2015). Therefore, phytocannabinoids have a more enriched pharmacological profile than modulating only cannabinoid receptors, and the number of molecular targets is steadily increasing. Notably, the phytocannabinoid CBD has drawn considerable attention as a treatment option for anxiety, PTSD, depression, autism, or schizophrenia (Fiani et al., 2020). CBD application in chronic pain and inflammation (Argueta et al., 2020), the common symptons in IBS, is also promising but more studies are necessary. The underlying mechanisms of CBD action are complex but include GPR12, and GPR3 and GPR6 (Brown et al., 2017; Morales et al., 2018; Laun et al., 2019), besides many others, such as GPR55, TRPV1, and serotonin 5-HT_1A_ receptor (Silvestro et al., 2020).

## The Intestinal Endocannabinoid System

### Expression of Endocannabinoid System Components in Gut Epithelium and Enteroendocrine Cells

**FIGURE 1:**
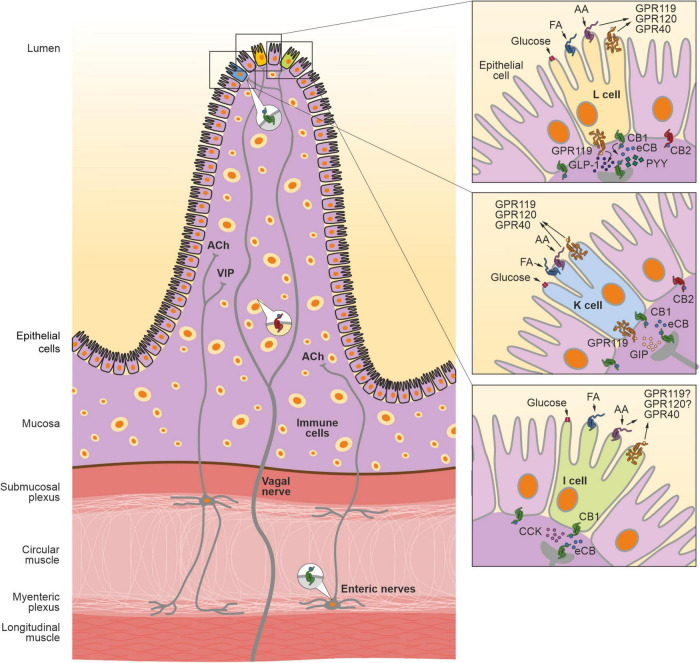
The endocannabinoid system in the gastrointestinal tract. The crosstalk between the intestinal eCB system and microbiota regulates many GI functions, such as gut permeability, motility, hormonal secretion, nutrient absorption and immune response. CB1R is expressed in different cells in the gut, including epithelial cells (colored in violet) and different types of enteroendocrine cells (colored in yellow, L cell; blue, K cell, and green, I cell, respectively) in the mucosa layer. In the myenteric and submucosa plexus, CB1R is present in the enteric nervous system, in particular, in the cell bodies of cholinergic neurons containing the neurotransmitter acetylcholine (ACh). Furthermore, CB1R is expressed in the afferent vagal neurons. CB2R is expressed in immune cells as well as in the epithelial cells in the mucosa. AA, arachidonic acid; CCK, cholecystokinin; FA, fatty acids; GIP, gastric inhibitory peptide; GLP-1, glucose-like peptide 1; GPR119, G protein-coupled receptor 119; GPR120, G protein-coupled receptor 120; GPR40, G protein-coupled receptor 40; PYY, peptide YY.

CB1R is localized in different components of the gut, such as epithelium, smooth muscle, submucosal myenteric plexus, and the myenteric ganglia (Wright et al., 2005; Grill et al., 2019a; Figure 1). In the myenteric ganglia, CB1R is found in cell bodies of the neurons expressing choline acetyltransferase (ChAT), calcitonin gene-related peptide (CGRP) and substance P (Kulkarni-Narla and Brown, 2000; Adami et al., 2002; Coutts et al., 2002). In contrast, CB2R is mainly localized in the plasma cells and macrophages in the GI mucosa and submucosa (Wright et al., 2005; Figure 1). CB2R is also expressed in intestinal epithelial cells in the GI mucosa (Rousseaux et al., 2007; Figure 1). eCB components are also found in the enteroendocrine cells, such as I-cells, K-cells, L-cells and enterochromaffin cells (Moss et al., 2012; Sykaras et al., 2012; González-Mariscal et al., 2016). Furthermore, the transcripts for GPR119 and CB1R are present in isolated I-cells, K-cells and L-cells (Overton et al., 2008; Sykaras et al., 2012; Moss et al., 2016; Figure 1). The AEA degrading enzyme fatty acid amide hydrolase (FAAH) is expressed in different regions and cells of the gut (Katayama et al., 1997; Pinto et al., 2002; Capasso et al., 2005). The small intestine and colon show high activity of FAAH, especially during gut inflammation (Izzo et al., 2001; Pinto et al., 2002; de Filippis et al., 2008).

Similarly, the 2-AG degrading enzyme, monoacylglycerol lipase (MAGL), is widely localized in the gut (Duncan et al., 2008; Grill et al., 2019a). MAGL is expressed in the mucosal and muscular layer of the ileum, duodenum, and colon. The highest activity of MAGL is reported in the duodenum, while lowest activity is observed in the distal part of the colon. MAGL is also expressed by the myenteric neurons, where colocalization mainly with the calretinin-positive neurons is reported, while no colocalization could be observed with the nitric oxide synthase (NOS) expressing neurons (Duncan et al., 2008). In mice, the 2-AG synthesizing enzyme diacylglycerol lipase α (DAGLα) is detected throughout the gut and enteric nervous system. It is prominently expressed by the myenteric plexus and is co-localized with ChAT. Moreover, inhibiting cholinergic activity in colon *ex vivo* by scopolamine increases 2-AG, while inhibiting DAGLα normalizes 2-AG level (Bashashati et al., 2015). Of note, both plant-derived cannabinoids and eCBs can bind and affect gut physiology through TRPV1-4 (De Petrocellis et al., 2012; Capasso et al., 2014). In the model of IBS, TRPV1 was found to be dysregulated, and the treatment with eCBs or phytocannabinoids restored its normal physiological function, highlighting the importance of this receptor in gut physiology and pathophysiology (De Petrocellis et al., 2012; Capasso et al., 2014). In addition to CB1R and CB2R, there are three orphan GPCRs, GPR3, GPR6 and GPR12, which share high percentage of sequence homology with cannabinoid receptors and bind to CBD (Laun and Song, 2017; Morales et al., 2018; Li et al., 2021). However, their expressions in the GI tract have not been investigated thoroughly and contrary results exist (Morales et al., 2018; Li et al., 2021).

### Endocannabinoid Levels in the Gut

Endocannabinoids are synthesized in different parts of the gut, and their levels change depending upon the metabolic and inflammatory status. In a sham feeding rat model, the levels of 2-AG and AEA increase after orosensory exposure of dietary fats in a vagus nerve-dependent manner. Interestingly, this effect was absent with the carbohydrate or protein intake (DiPatrizio et al., 2011). Metabolic conditions can influence eCB levels; fasting increases, while re-feeding decreases AEA and 2-AG levels in the small intestine (Izzo et al., 2009; DiPatrizio et al., 2015). In obese human subjects compared to lean and overweight control, elevated levels of fasting plasma AEA are observed while duodenal expression of gap junction proteins, intestinal alkaline phosphatase (IAP) and zonula occludens (ZO-1), decrease and correlate negatively with the plasma AEA levels (Little et al., 2018). In rodents, increased obesity is associated with the increased levels of 2-AG and AEA in the duodenum both during fasting and refeeding (Izzo et al., 2009). Thus, the metabolic status can affect the gut eCB levels and gut permeability. Similarly, during gut inflammation, such as ulcerative colitis and in murine models of colitis, colonic AEA levels were elevated, while 2-AG levels were not different as compared with the control group (D’Argenio et al., 2006). Furthermore, the AEA reuptake inhibitor VDM-11 increases AEA levels and reduces inflammation in the colon of dinitrobenzene sulfonic acid-treated mice (D’Argenio et al., 2006). Contrarily, in intestinal inflammation induced by croton oil, no differences in the levels of 2-AG and AEA are observed in the small intestine of control and treated mice. However, the upregulation of CB1R expression is observed in croton-oil induced inflammation. Interestingly, inflamed small intestine showed two-fold higher activity of the AEA degrading enzyme anandamide amidohydrolase (Izzo et al., 2001). In addition, inhibition of FAAH by arachidonoyl serotonin (AA-5-HT) increases intestinal AEA and PEA levels in mice (Capasso et al., 2005).

### Regulation of the Gut-Barrier Function (Motility, Permeability, Nutrient Absorption) and the Endocannabinoid System

AEA, 2-AG and PEA are present in the colon and small intestine and have been shown to tonically inhibit the colonic propulsion in mice (Capasso et al., 2001; Izzo et al., 2001; Pinto et al., 2002). However, the effect of PEA on intestinal transit is apparently independent of CB1R activation, as the CB1R antagonist SR141716A fails to normalize the gut motility (Capasso et al., 2001). Similarly, in humans, AEA and 2-AG tonically inhibit cholinergic contractility of colonic longitudinal and circular muscles independent of cannabinoid receptor-mediated pathways (Smid et al., 2007). Contrarily, the inhibition of MAGL activity decreases gut transit in a CB1R dependent manner, as the effect is not observed in CB1R deficient mice (Duncan et al., 2008; Taschler et al., 2015). Furthermore, the genetic ablation of MAGL increases 2-AG levels, however, does not affect the gut transit time, possibly owing to desensitization of CB1R as evidenced by the increased immunohistochemical localization of CB1R in endocytic vesicles (Taschler et al., 2015). The pharmacological inhibition of DAGLα decreases the intestinal contractility and extends the whole gut transit time (Bashashati et al., 2015). Most of the effects of eCBs on gut motility are mediated by ChAT. However, there are several other neurotransmitters known to affect gut motility, which can be modulated by the gut eCB system. AEA decreases while CB1R blockade increases both, the ascending contraction, and the descending relaxation, by modulating the levels of substance P and vasoactive intestinal peptide (VIP) (Grider et al., 2009). This study further confirms that AEA decreases while the CB1R antagonist AM251 increases the release of CGRP. Thus, eCBs can inhibit the gut motility both by inhibiting the excitatory cholinergic neurons and inhibitory VIP motor neurons as well as by inhibiting the CGRP neuron-mediated initiation of peristaltic reflex (Grider et al., 2009).

Gut permeability and nutrients absorption are modulated during various physiological and pathophysiological conditions. The pathological conditions, such as obesity, diabetes, and the inflammation of the gut, affect gut permeability and nutrient absorption. 2-AG and AEA, in addition to its role in gut motility, affect gut permeability, thereby regulating the nutrient absorption. The dysregulation of the eCB system in gut is observed in various pathophysiological and inflammatory conditions. Inflammatory bowel diseases (IBD) is associated with the impaired epithelial barrier function and increased gut eCB system activity (Gassler et al., 2001; Massa et al., 2004; Bruewer et al., 2006; D’Argenio et al., 2006; Amasheh et al., 2009). Interestingly, eCBs and phytocannabinoids have been shown to exert opposite effects on gut permeability. The eCBs 2-AG and AEA increase the permeability associated with the inflammation, while phytocannabinoids such as THC and CBD restore the permeability increased by cytokines (Alhamoruni et al., 2010, 2012). In the cell culture model, AEA and 2-AG increase the permeability of human colorectal adenocarcinoma (Caco-2) cell monolayer and synergize with the cytokines to enhance the permeability in a concentration dependent manner (Alhamoruni et al., 2010). More recently, Caco-2 cell permeability induced by hypoxia can be modulated by eCBs, thus presenting a novel therapeutic target against gut disorders caused by the increased permeability (Karwad et al., 2019).

### Regulation of the Gastrointestinal Hormone Secretion and the Endocannabinoid System

The eCB system is present in the enteroendocrine cells secreting GI hormones (Moss et al., 2012; Sykaras et al., 2012; González-Mariscal et al., 2016). Enteroendocrine cells are spread across the gut and secret GI hormones, such as incretins, peptide YY, cholecystokinin (CCK), somatostatin, VIP, gastrin etc. In addition, enterochromaffin cells secrete neurotransmitters including serotonin, which regulate gut motility and act on afferent and efferent nerves of the enteric nervous system to modulate the gut-brain axis (Matthes and Bader, 2018). CB1R mRNA and immunoreactivity have been detected in CCK-secreting I-cells (Sykaras et al., 2012) (Figure 1). Indeed, eCB signaling in the small intestine has been implicated in the suppression of meal-induced CCK release in obesity (Argueta et al., 2019). Furthermore, 2-AG directly stimulates CCK secretion in murine enteroendocrine STC-1 cells (Ochiai et al., 2021). Therefore, eCBs can directly control the nutrient-induced release of CCK. In addition to I-cells, CB1R is expressed in K- and L-cells in the small intestine of rodents (Moss et al., 2012) (Figure 1). However, CB1R could not be detected in L-cells from the colon (Moss et al., 2012). K-cells synthesize and secrete gastric inhibitory peptide (GIP), while L-cells secrete glucacon-like peptide 1 (GLP-1) in response to nutrient ingestion.

Recently, in obese humans, nabilone, a synthetic cannabinoid, increases both fasting and post-glucose intake GIP levels while reduces post-glucose intake GLP-1 levels (Chia et al., 2017). In contrast, in rodents, prior administration of methanandamide suppresses GIP release in the oral glucose tolerance test (OGTT), but does not affect GLP-1 levels, while CB1R antagonist administration increases GIP levels (Moss et al., 2012). Furthermore, *in vitro* studies performed on murine K-cells confirm the inhibitory effect of methanandamide on GIP release (Moss et al., 2012). In addition to classical cannabinoid receptors, GPR119 transcripts have been detected in L-cells. OEA has been shown to increase GLP-1 secretion, both *in vitro* and *in vivo*, from intestinal L-cells by acting through GPR119 (Overton et al., 2006; Lauffer et al., 2009). GPR55 has been shown to affect energy homeostasis, adipogenesis and insulin secretion (Lipina et al., 2019; Ramírez-Orozco et al., 2019). Interestingly, GPR55 is expressed in gut epithelium and the enteric nervous system, suggesting its neuroendocrine role in controlling gut physiology (Schicho et al., 2011; Schicho and Storr, 2012). The phytocannabinoid, CBD, can antagonize GPR55 and, this explains some of the discrepancies observed in eCB treatments (Schicho and Storr, 2012; Laprairie et al., 2015). In addition, CB1R is located on vagal afferent neurons and enterochromaffin cells, suggesting its role in gut neurotransmitter release and gut-brain axis (Burdyga et al., 2004; Ye et al., 2021). Notably, enterochromaffin cells contribute to 95% of the peripheral serotonin and regulate GI motility (Spiller, 2001; Liu et al., 2021).

### Microbiota and the Intestinal Endocannabinoid Tone

The roles of microbiota in gut physiology and gut brain-axis have been convincingly established. The role of microbiota in modulating intestinal eCB tone was first revealed by Rousseaux et al. (2007), who showed that the bacterium *Lactobacillus acidophilus* when administered orally in mice and rats increases the expression of intestinal epithelial CB2R. Furthermore, *Lactobacillus acidophilus* stimulates CB2R mRNA expression in resting human HT-29 epithelial cells, confirming the role of gut microbiota in modulating the intestinal eCB tone (Rousseaux et al., 2007). The role of gut microbiota in intestinal eCB tone regulation was further confirmed by manipulating the gut microbiota by various means, such as antibiotic treatment, probiotic treatment, high fat diet (HFD), and by mutations in the Myd88 gene which disrupt TLR-mediated bacteria-host interaction (Everard et al., 2014; Guida et al., 2018; Castonguay-Paradis et al., 2020). In all these models, changes in the abundance of CB1R mRNA were observed in the colon, whereas CB1R mRNA levels in the jejunum remained unaffected. Similarly, FAAH and MAGL levels in the colon were modulated by changes in gut microbiota (Muccioli et al., 2010). However, CB2R mRNA levels remained unaffected in these models, suggesting that the gut microbiota selectively upregulate CB1R in colon (Muccioli, 2010). Contrarily, another study showed the up-regulation of colonic CB2R in dysbiotic mice, but the down-regulation of colonic CB1R (Aguilera et al., 2015).

Recently, *Akkermansia muciniphila* has been identified as an important bacterium regulating the gut eCB tone, gut permeability and secretion of gut peptides (Everard et al., 2013). *Akkermansia muciniphila* treatment increases the levels of 2-OG, 2-AG, and 2-PG in the intestine and reverses the HFD-induced metabolic dysregulation (Everard et al., 2013). Furthermore, the oral treatment with CB1R antagonist SR141716A increases *Akkermansia muciniphila*, and decreases *Lachnospiraceae* and *Erysipelotrichaceae* in obese mice compared to control group (Mehrpouya-Bahrami et al., 2017). Similarly, dysbiosis induced by a cocktail of antibiotics decreases AEA level in the duodenum, while probiotic treatment significantly increases AEA level in the jejunum (Guida et al., 2018). The probiotic treatment reduces OEA level, while does not affect PEA and 2-AG levels (Guida et al., 2018). Thus, the microbiota can regulate the intestinal eCB tone. Conversely, the changes in intestinal eCB tone might affect the microbiota composition. MAGL deficient mice show higher levels of *Ruminococcus*, *Rose buria*, and *Hydrogenoanaerobacterium* than wild type mice (Dione et al., 2020). These alterations in the gut microbiota were mainly due to changes in 2-AG level which were further confirmed, *in vitro*, by the culture of fecal microbiota in the presence of 2-AG (Dione et al., 2020). The increased eCB levels can also modulate the host susceptibility to infections. Some studies suggest that the bacterial genomes code for lipases that can hydrolyze 2-AG *in vitro* (Côtes et al., 2007; Dhouib et al., 2010). A recent study suggests that mice with increased 2-AG levels are protected from *Enterobacteriaceae* pathogens (Ellermann et al., 2020). The murine attaching and effacing pathogens such as *Citrobacter rodentium* or *Salmonella typhimurium* are less virulent in mice with increased 2-AG levels, owing to the antagonizing effect of 2-AG on the bacterial receptor QseC and the subsequent activation of type III secretion systems (T3SS). This study further confirms that the genetic ablation of MAGL renders mice less susceptible to intestinal diseases caused by *Citrobacter rodentium* (Ellermann et al., 2020). Therefore, a close and bidirectional relationship between gut microbiota and gut eCB tone exists, and thereby can modulate each other.

Further studies from germ-free (GF) mice confirm the effect of microbiota on intestinal eCB tone. The expression of CB1R, GPR18, GPR55 and PPARα in the duodenum, jejunum, ileum, and proximal colon of GF mice show significant differences compared to conventionally raised mice (Manca et al., 2020). The expression of CB1R in the ileum of GF adult mice increases by twofold, which can be partially reversed by the transfer of feces from conventionally raised mice (Manca et al., 2020). However, the expression of the orphan G-coupled receptors GPR55 and GPR18 decreases in the small intestine of the both young and adult GF mice except in the duodenum of young GF mice (Manca et al., 2020). The eCBs levels also showed significant changes in the small intestine of GF mice. The AEA levels were found significantly elevated in jejunum and colon of adult GF mice (Manca et al., 2020).

### Role of the Intestinal Endocannabinoid System in Obesity and Diabetes

Obese and diabetic leptin resistant (db/db) mice show changes in the microbiome composition, eCB tone, and inflammation (Geurts et al., 2011). HFD-fed obese mice show threefold higher CB1R mRNA levels in the colon, which decrease after probiotic treatment (Muccioli et al., 2010). The role of gut eCBs in diabetes has also been envisaged given the role of eCBs in regulating the intestinal permeability. In fact, eCBs can regulate the circulating bacterial lipopolysaccharide (LPS) levels by modulating the gut permeability, which is associated with a low-grade inflammation, insulin resistance and obesity (de La Serre et al., 2010; Muccioli et al., 2010). Conversely, metabolic endotoxemia induced by the LPS infusion causes obesity and insulin resistance (Cani et al., 2007). Consequently, the antibiotic treatment reduces gut inflammation and the cecal content of LPS, which is also associated with a sharp reduction in the colonic CB1R levels (Cani et al., 2008; Muccioli et al., 2010). In addition, the gut eCB system can control dietary fat intake and food preference. In mice, treatment with the CB1R antagonist AM251 or deletion of CB1R from intestinal epithelium inhibits the food preference for Western diets (Avalos et al., 2020). Furthermore, fat feeding, particularly the dietary unsaturated fats, increases 2-AG and AEA levels in the jejunum. Consequently, the local infusion of the CB1R antagonist rimonabant into the duodenum inhibits fat intake, an effect mediated through the vagus nerve and the gut-brain axis (DiPatrizio et al., 2011). Recently, it was shown that the genetic deficiency of MAGL reduces the intestinal absorption of fats by a mechanism independent of CB1R, as the phenotype persists even in the CB1R/MAGL double deficient mice (Yoshida et al., 2019). Interestingly, MAGL deficient mice show less preference for HFD over normal chow. Moreover, the oral administration of lipids suppresses appetite both in the MAGL deficient and CB1/MAGL double deficient mice compared to WT control and CB1 KO mice, the effect which requires the intact gut-brain communication through the vagus nerve (Yoshida et al., 2019). High fat and palatable diet alter the intestinal eCB system and microbiome composition (Lacroix et al., 2019; Dione et al., 2020). The palatable diet increases 2-AG and AEA levels in plasma and affects the relative abundance of several intestinal microbiota. Furthermore, a weight-independent correlation exists between the relative abundance of microbiota and the AEA levels (Lacroix et al., 2019). Everard et al. (2013) showed that the abundance of *Akkermansia muciniphila* is 3300-fold lower in leptin deficient (ob/ob) mice and its abundance decreases by 100-fold in HFD-fed mice. Accordingly, the treatment with this bacterium increased intestinal 2-AG, 2-OG, and 2-PG levels (Everard et al., 2013). Similar to HFD, vitamin D deficiency can also affect the gut eCB tone by modulating the microbiota composition and the levels of AEA and 2-AG in duodenum and colon (Guida et al., 2020). In humans, obesity is associated with the increased AEA levels and reduced expression of ZO-1 and IAP, the gap junction proteins (Little et al., 2018). Furthermore, switching from Western diet to isocaloric Mediterranean diet decreases plasma 2-AG levels, increases plasma OEA and PEA levels, and increases fecal *Akkermansia muciniphila* abundance, independent of the changes in body weight (Tagliamonte et al., 2021).

### The Endocannabinoid System and Inflammatory Bowel Disease

IBD, including Crohn’s disease and ulcerative colitis, has been associated with the chronic inflammation and the perturbed microbiota composition in gut (Jalanka-Tuovinen et al., 2014; Zhang et al., 2014; Grill et al., 2019b). However, it is not clear whether the inflammation is caused by the changes in gut metabolites and gut microbiota, or the changes in gut microbiome is a consequence of IBD. In IBD, Firmicutes, Proteobacteria, Verrucomicrobia, and Fusobacteria were abundant, while Bacteroidetes and Cyanobacteria decreased as compared to healthy subjects (Santoru et al., 2017). The eCB system has been implicated in IBD pathophysiology (Hasenoehrl et al., 2017; Grill et al., 2019b). Furthermore, several studies have confirmed the therapeutic potential of modulators of eCB biosynthesis and degradation in IBD (Alhamoruni et al., 2012; Hasenoehrl et al., 2017). The pharmacological inhibition of FAAH has been shown to reduce inflammation and to promote gut healing (Sasson et al., 2006). Cannabinoid receptors also mediate the low-carbohydrate diet-induced improvement in the gastro-intestinal function and provide protection to the intestinal crypt base (Gigante et al., 2021). In the mouse model of chemical-induced colitis, CB1R protects from the pro-inflammatory responses, while the genetic deficiency of CB1R induces stronger inflammatory responses (Massa et al., 2004). In contrast, chemical-induced colitis increases the percentage of CB1R-expressing myenteric neurons, while the cannabinoid receptor agonist HU210 or the genetic ablation of FAAH protects from the chemical-induced colitis (Massa et al., 2004). Similarly, mustard oil-induced transient colitis and inflammation is found to be associated with the accelerated transit of upper GI tract, which is accompanied by an increase in the levels of intestinal AEA and decrease in the TRPV1 mRNA levels (Capasso et al., 2014). However, no increase is observed in the levels of 2-AG, PEA, or OEA.

In patients with ulcerative colitis and Crohn’s disease, vulnerability to disease is modulated by the polymorphism 1359G/A in the CB1R gene, confirming the role of the eCB system in IBD (Storr et al., 2010; Sharkey and Wiley, 2016). The role of CB1R in gut motility and gut-brain axis is further confirmed by the genetic variants of the CB1R gene. In the Korean population, allele frequencies of AAT triplet repeat in the CB1R gene were different between the normal and IBS patients (Park et al., 2011). This finding was further confirmed in the Chinese population, showing the susceptibility to IBS in patients with the longer alleles of AAT triplet repeat (Jiang et al., 2014). Contrarily, this association could not be established in the Caucasian population (Camilleri et al., 2013). However, contrary results exist about the polymorphism in FAAH gene (Camilleri et al., 2013; Jiang et al., 2014). Therefore, apparently, there might be ethnic differences in the genetic polymorphism in eCB system related genes.

### Endocannabinoid System and Microbiota in Gastrointestinal Cancer

Gastrointestinal cancer is often associated with the chronic gut inflammation, which is an important risk factor in cancer initiation and tumor invasion. Several studies have elucidated the link between dysregulated eCB system, chronic gut inflammation and cancer (Hermanson and Marnett, 2011; Lee et al., 2016; Cherkasova et al., 2021; Hryhorowicz et al., 2021). CB1R and CB2R agonists, FAAH inhibitors, and phytocannabinoids have been shown to reduce gut inflammation (Massa et al., 2004; Kimball et al., 2006; Storr et al., 2009; Alhouayek et al., 2011; Borrelli et al., 2013; Sałaga et al., 2014). In addition, PEA and AEA have been shown to reduce gut and systemic inflammation (D’Argenio et al., 2006; Esposito et al., 2014). Therefore, the microbiota mediated dysregulation of eCBs and concomitant chronic gut inflammation can be a causative link for GI cancer. Gut microbiota such as *Akkermansia muciniphila* is known to increase the levels of 2-AG, 2-OG, and 2-PG in HFD fed mice, and thus can have a protective effect against gut inflammation and GI cancer (Everard et al., 2013).

Due to increased consumption of Western diets, a rapid increase in esophageal adenocarcinoma has been observed mainly owing to obesity and gastroesophageal reflux disorder. Certain microbiota such as *Actinobacillus*, *Campylobacter*, *Prevotella*, *Streptococcus*, *Veillonella*, and *Leptotrichia* are found enriched in the Barrett esophagus, a pre-cancerous lesion. In esophageal squamous cell carcinoma, the most abundant species are *Porphyromonas gingivalis*, and *Veillonella parvula* while *Peptococcus, Moryella, Corynebacterium, Catonella, Lautropia*, *Bulleidia, Moryella*, *Peptococcus*, *Cardiobacterium*, and *Treponema* genus is depleted (Chen X. et al., 2015; Liu et al., 2018; Smet et al., 2022). In human patients with esophageal carcinoma, CB1R gene mutation has been shown to affect tumor susceptibility (Bedoya et al., 2009). The main gut microbiota which has been implicated in the gastric cancer is *Helicobacter pylori* and it is suspected to facilitate and prime the gastric mucosa for carcinogenesis, as it is not found in the adrenocarcinoma stage (Ferreira et al., 2018). In a recent study, human gastric adenocarcinoma cell line, AEA has been shown to attenuate the inflammasome activation and reduce IL-1β level through the activation of CB1R and TRPV1 (Sedeighzadeh et al., 2021). Notably, the inflammasome activation and IL-1β secretion are involved in gastric inflammation and the development of the gastric cancer. The inhospitable pH of the stomach makes difficult for other bacteria to inhabit. However, with the use of advanced techniques, microbiota such as Firmicutes, Bacteroidetes, Proteobacteria, Actinobacteria, and Fusobacteria have been detected in the stomach (Eun et al., 2014). Contrary to gastric cancer, numerous microbial species have been involved in colorectal cancer. In the colon, some gut microbiota have protective roles against cancer, while others are pro-oncogenic. The microbiota producing SCFAs, such as acetate, butyrate, and propionate, have a protective role against cancer. Butyrate has been shown to have a strong preventive and therapeutic effects on cancer in mouse and cellular models (Donohoe et al., 2014). The eCB system has been implicated in colorectal cancer, and elevated levels of eCBs are found in adenomas as compared to healthy control (Zaiachuk et al., 2021). However, increased eCB levels might reduce the expression of CB1R (Wang et al., 2008). The level and the activity of the enzymes involved in the biosynthesis of AEA, NAPE-PLD, were upregulated in patients with colorectal cancer (Chen L. et al., 2015). A recent study suggests that during inflammation or carcinogenesis, the expression of CB1R decreases in enterocytes due to epigenetic changes in CB1R promoter region (Zhang et al., 2017). The methylation in the CB1R gene has been positively correlated with the progression of colorectal cancer affecting the tumor size, depth of invasion, and tumor stage. Furthermore, immunohistochemical analysis of colorectal cancer samples shows more than 76% decrease in CB1R expression (Jung et al., 2013). Contrary to this, the activation of GPR55, has been shown to promote tumor growth in various types of cancer and alters the populations of myeloid-derived suppressor cells and T lymphocytes within the tumor tissues (Hasenoehrl et al., 2018). Moreover, tumor tissues from GPR55 KO mice show reduced expression levels of tumorigenic factors COX-2 and STAT3 (Hasenoehrl et al., 2018). Interestingly, methylation in the CB1R and GPR55 genes is also differentially regulated in human colorectal tissues. In a recent study, colorectal cancer patients with metastasis have shown a low expression of CB1R (Tutino et al., 2019). Interestingly, CB1R downstream signaling pathways were also altered in the normal mucosa surrounding the tumor, suggesting a molecular mechanism underlying malignant transformation. As discussed in detail in the previous section, the gut microbiota interacts with the intestinal eCB system to regulate gut permeability and inflammation. Therefore, dysbiosis and dysregulation of the intestinal eCB system can promote the pathological conditions which increase the susceptibility to colon cancer.

### Exogenous Cannabinoid Effects on Intestinal Physiological Functions and Possible Therapeutic Applications

Several studies have suggested the therapeutic potential of synthetic and plant-derived cannabinoids, however, the lack of sufficient preclinical data, often compounded with the multiple receptors mediating their effects, are major obstacles for their clinical use (Di Marzo and Piscitelli, 2015; Hryhorowicz et al., 2021). Multiple studies have confirmed the role of both exogenous cannabinoids and eCB signaling in providing protection against the gut inflammation in IBD (Naftali et al., 2013; Storr et al., 2014; Fraguas-Sánchez and Torres-Suárez, 2018; Grill et al., 2019b). The studies using CB1R and CB2R agonist, and the inhibitors of MAGL and FAAH have shown encouraging results in mitigating gut inflammation in rodent models of IBD (Massa et al., 2004; Kimball et al., 2006; Storr et al., 2009; Alhouayek et al., 2011; Sałaga et al., 2014). In addition, phytocannabinoids such as CBD can reduce gut inflammation through GPR55 (Stančić et al., 2015; Pagano et al., 2016). Only limited preclinical studies in humans using cannabis for the treatment of IBD are available due to legality issues associated with its use. However, a few studies available on humans do not suggest any drastic improvement in the inflammatory markers, however, most of the patients using cannabis report improvement in the discomfort and pain associated with IBD (Picardo et al., 2019; Kienzl et al., 2020). CBD has been shown to have therapeutic potential in neurodegenerative disorders (Cassano et al., 2020). PEA is an endogenously produced eCB-like compound, which has been shown to have therapeutic potential in neurodegenerative disorders, inflammatory diseases, and pain perception (Petrosino and Di Marzo, 2017). Cannabis extracts and THC, when administered orally, have been shown to affect gut motility by modulating the cholinergic transmission (Chesher et al., 1973; Roth, 1978; Coutts and Pertwee, 1997), an effect which is attributed to CB1R (Yuece et al., 2007). Later, several other synthetic cannabinoids were shown to modulate emesis, suggesting its possible therapeutic use (Sharkey et al., 2014). Cannabis extract rich in CBD inhibits colorectal cancer cell proliferation through CB1R and CB2R (Romano et al., 2014; Raup-Konsavage et al., 2018). However, CB2R has been found to be a poor prognostic marker, raising the question about its therapeutic use (Martínez-Martínez et al., 2015).

Despite the therapeutic potential of exogenous cannabinoids in several gastro-intestinal diseases in rodent models, their therapeutic use has not yet been approved in humans. Recently, the US Food and Drug Administration (FDA) has approved CBD for the therapeutic use in cancer and AIDS patients to treat the nausea associated with cancer chemotherapy and the anorexia associated with AIDS, respectively, (Cooper et al., 2021). However, more clinical studies for the use of cannabinoids in humans are required.

## The Gut-Brain Axis and the Endocannabinoid System

### Bidirectional Gut-Brain Axis

**FIGURE 2:**
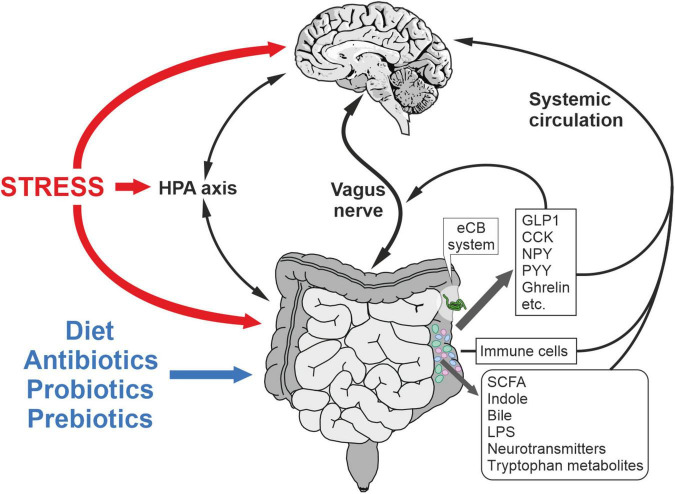
Microbiota-gut-brain axis in the regulation of stress responses and metabolism. Mechanisms underlying the bidirectional communication include the vagus nerve, immune system, circulating mediators and HPA axis. Microbiota composition is affected by stress, diet, prebiotics, postbiotics and antibiotics among other factors. CCK, cholecystokinin; GLP-1, glucagon-like peptide-1; LPS, lipopolysaccharide; NPY, neuropeptide Y; PYY, polypeptide YY; SCFA, short chain fatty acid.

Multiple studies have convincingly established that the changes in gut microbiota induced by antibiotic, probiotic and GF conditions can lead to behavioral dysregulation related with emotion, anxiety, and stress coping. Conversely, stress and neuropsychiatric disorders can perturb the microbiota composition. Therefore, this strongly suggests the existence of a bidirectional communication between gut and brain, called the microbiota-gut-brain axis (Figure 2). Many studies suggest the role of gut neurotransmitters and hormones in the modulation of host-bacteria interaction and virulence. Host’s neurotransmitters can affect the colonization pattern and virulence of gut microbiota (Kendall and Sperandio, 2016). Furthermore, the presence of interaction sites for these hormones on bacteria has been shown (Hughes and Sperandio, 2008). A receptor for epinephrine/norepinephrine is present in enterohemorrhagic *Escherichia coli*, which is a membrane bound histidine sensor kinases (HKs) and can be blocked by an α-adrenergic antagonist (Clarke et al., 2006; Reading et al., 2009). HKs have both kinase and phosphatase activity, and cellular signals can either activate or deactivate. Similarly, a strain of *Pseudomonas fluorescens* was shown to possess a receptor for γ-aminobutyric acid (GABA) with binding characteristics similar to GABA receptors found in the brain, which can be inhibited competitively by the GABA analog muscimol (Guthrie and Nicholson-Guthrie, 1989). Recently, the highly abundant neurotransmitter serotonin, which is also secreted from enterochromaffin cells in the gut lumen, was shown to decrease the virulence of enterohemorrhagic *Escherichia coli* and *Citrobacter rodentium* in rodents. The binding site for serotonin is a membrane-bound histidine sensor kinase, CpxA, which inactivates the transcription factor CpxR after dephosphorylation induced by serotonin, controlling the virulence gene. Interestingly, CpXA is also expressed by other gut bacteria. Thus, serotonin can affect the microbiota composition and virulence (Kumar et al., 2020).

Conversely, microbiota composition can affect the release of gut hormones and neurotransmitters and can communicate with the brain via the vagus nerve (Figure 2). Studies using GF mice suggest deficient circulating and colon serotonin levels as compared to specific-pathogen free (SPF) mice (Wikoff et al., 2009; Yano et al., 2015). The gut microbiota can regulate serotonin synthesis both in mice and humans. In GF mice, the colonization of spore-forming bacteria increases serum serotonin and the colonic expression of tryptophane hydroxylase 1 (TPH1) (Yano et al., 2015). Interestingly, the colonization of GF mice with spore-forming bacteria from human colonic microbiota yields the same effects on serotonin levels, thus suggesting that the effects of these microbes on serotonin synthesis is conserved across mice and humans (Yano et al., 2015). Enteroendocrine cells respond to gut metabolites and modulate the secretion of GI hormones, thereby regulating food intake, satiety, and gut motility (Wu et al., 2013). Gut hormones and neuropeptides, such as CCK, PYY and GLP-1 through its binding sites on the vagus nerve, communicate with the brain to regulate behavioral and metabolic processes. The vagus nerve mediates the bidirectional communication between the brain and the gut microbiota (Bellono et al., 2017; Bonaz et al., 2018; Figure 2). Recently, it has been shown that LPS-induced depression-like phenotype and alterations in the gut microbiota diversity are not observed after subdiaphragmatic vagotomy, indicating the vagus nerve-mediated communication between the gut and the brain (Zhang et al., 2020). Several earlier studies also confirmed that oral administration of intestinal bacteria, such as *Campylobacter jejuni* and *Lactobacillus rhamnosus*, induces c-fos activation in brainstem regions receiving afferent sensory information from the GI tract and produce region-specific changes in GABAB1b receptor expression in the mouse brain (Gaykema et al., 2004; Goehler et al., 2005; Bravo et al., 2011). Furthermore, these changes can lead to stress-induced corticosterone (CORT) release, and anxiety and depression-like behavior. These effects are mediated by the vagus nerve, as resection of the vagus nerve abolishes these effects, suggesting a constitutive communication between brain and gut via the vagus nerve (Bercik et al., 2011b; Bravo et al., 2011). In addition, oral antimicrobials increase hippocampal BDNF in SPF mice, independent of the inflammation, vagal or sympathetic system. Interestingly, this effect was absent when antimicrobials were administered intraperitoneally, suggesting the intermediary role of neurally active metabolites secreted from the gut microbiota (Bercik et al., 2011a).

Microbial metabolites, such as indole, produced by the gut microbiota through the enzyme tryptophanase have been shown to modulate the gut-brain axis (Kumar and Sperandio, 2019; Figure 2). GF rats, when transplanted with bacterial species overproducing indole, exhibit increased anxiety-like behavior, which is primarily due to the increased vagus nerve activation (Jaglin et al., 2018). In mice, chronic indole production does not cause behavioral changes, but it does affect behavior under duress and increases the susceptibility of male mice to chronic mild stress (Mir et al., 2020). Furthermore, this study showed that indole increases the expression of the phenylethanolamine *N*-methyltransferase (PNMT) gene in the adrenal medulla and thus affects the synthesis of noradrenaline. The SCFAs, such as the acetate, propionate, and butyrate, are also produced by microbiota in the colon, which have been implicated in the behavior and several neurophysiological disorders (Silva et al., 2020; Figure 2).

### Vagus Nerve

The vagus nerve acts as a bidirectional communication pathway between the periphery and the brain and is one of the principal components in the microbiota-gut-brain axis (Mayer, 2011; Prescott and Liberles, 2022; Figure 2). Importantly, the GI tract is highly innervated by both efferents and afferents of the vagus nerve. The efferent vagal preganglionic fibers from the dorsal motor nucleus vagus (DMV) innervate postganglionic neurons located in the myenteric and submucosal plexus of the gut (Berthoud and Neuhuber, 2019). Furthermore, the vagus afferents include the primary sensory neurons that convey both mechanical and chemical information from the GI tract to the brain (Han et al., 2018; Bai et al., 2019). The cell bodies of these sensory neurons are located in the nodose ganglia, which send bipolar axons to both the GI tract and the nucleus tractus solitarius (NTS). From the NTS, the vagal nerve can regulate via few synapses the neuronal activity of e.g., forebrain regions, importantly those ones involved in reward as well as aversion (Mayer, 2011; Han et al., 2018). Han et al. (2018) elegantly established the gut-to-brain neuronal circuit that links vagal sensory neurons to the reward circuit controlling the striatal dopamine release. They mapped the neuronal circuit that connects the right vagal nodose ganglia to nigral dopamine neurons using an anterograde transynaptic viral tracer. The mapping approach elucidated a reward circuit consisting of the right nodose ganglia, the parabrachio-nigral pathway and its target in the dorsal striatum (Han et al., 2018). Similarly, as postulated previously (Craig, 2002), the vagal afferents might influence the regulation of emotional responses by sending interoceptive inputs via NTS-parabrachial nucleus-thalamus pathway to insula cortex (Mayer, 2011). The insula cortex (also referred as interoceptive cortex) is an integrative hub that is highly connected to amygdala, ventral striatum and prefrontal cortex (Gehrlach et al., 2020), all these brain regions have a pivotal role in stress-related affective states. Notably, it has been reported that CB1R is expressed in vagal afferents innervating different regions of the GI tract (Vianna et al., 2012; Egerod et al., 2018; Berland et al., 2022; Figure 1). Overall, the vagal nerve is a critical component of the microbiota-gut-brain axis in the regulation of food intake, hedonic feeding, visceral pain, aversion and emotions, but the anatomical mapping of the underlying circuits is still poorly understood.

### Gut Microbiota

Dietary components and specific amino acids, such as glutamine and tryptophan, affect the crosstalk between microbiome and gut permeability (Chakaroun et al., 2020; Massier et al., 2021). The gut permeability can be modulated by microbial metabolites such as SCFAs produced from the metabolism of dietary fibers (Chen et al., 2017). In rodents, HFD alters gut microbiota, and bile acid metabolism, leading to increased levels of inflammatory cytokines and increased gut permeability (Stenman et al., 2012; Hamilton et al., 2015). Furthermore, prolonged dietary intake of HFD decreases tight junction proteins expression (claudin-1, claudin-3, occluding and junctional adhesion molecule-1) in small intestine (Suzuki and Hara, 2010). The gut microbiota control the intestinal eCB tone and therefore, affect gut motility and gut permeability. CB1R blockade improves gut barrier function and alters the levels and distribution of ZO-1 and occluding gap junction proteins, while increasing eCB tone enhances the gut permeability and increase plasma LPS levels (Muccioli et al., 2010). The allogenic transplantation of fecal microbiota from lean donor to male recipient with metabolic dysregulation improves insulin sensitivity and affects plasma level of GABA (Kootte et al., 2017). Importantly, serotonin secretion from enterochromaffin cells is microbiota-dependent and therefore, gut motility can be modulated by gut microbiota by altering the levels of neurotransmitter release from the enteric nervous system (Yano et al., 2015). The analysis of enteroendocrine cell number and distribution in SPF and GF mice suggests the upregulation of enteroendocrine cell numbers, specifically the enterochromaffin cells, K-cells and L-cells (Modasia et al., 2020). Thus, the microbiota can affect the enteroendocrine cells and the release of neurotransmitters from the enteric neurons (Woźniak et al., 2021).

### Gut-Brain Axis Effects on Whole-Body Metabolism, Metabolic Disorders, and Inflammation

The microbiota composition in human subjects with metabolic disorders, such as type 2 diabetes, obesity and associated metabolic complications including hyperlipidemia, atherosclerosis, and hepatic steatosis, differs from that of healthy humans, suggesting its role in whole-body metabolism (Turnbaugh et al., 2006; Kootte et al., 2012; Gregory et al., 2015; Walker et al., 2021; Bielka et al., 2022). GF mice have less total body fat and body weight gain compared to SPF mice despite having higher food intake, thus confirming the role of microbiota in metabolic regulation (Turnbaugh et al., 2006). The effect of microbiota on host metabolism is mediated by the gut-brain axis and microbial metabolites, affecting gut permeability, inflammation, and insulin resistance. Consequently, dysbiosis induced by antibiotics reduces the metabolic endotoxemia and LPS levels in obese mice, leading to improved metabolic profile (Cani et al., 2008; Figure 2). The gut eCB system has been implicated in gut inflammation and metabolic regulation. CB1R antagonists affect gut microbiota composition, reduce inflammation and M1 macrophages in adipose tissue and plasma LPS level, and therefore reduce intestinal permeability and metabolic endotoxemia (Mehrpouya-Bahrami et al., 2017). Furthermore, CB1R blockade increases the abundance of *Akkermansia muciniphila* in the gut, while decreases *Lachnospiraceae* and *Erysipelotrichaceae*, confirming the eCB system-mediated changes in microbiota composition (Mehrpouya-Bahrami et al., 2017). In addition, engineered NAPE-expressing *Escherichia coli* bacteria, when administered through drinking water, reduces the adiposity in HFD fed mice by reducing food intake, improving insulin sensitivity and reducing hepatosteatosis (Chen et al., 2014). Thus, manipulating gut eCB signaling by using engineered gut bacteria might have therapeutic potential. In humans, gut microbiota transfer from lean to obese individuals improves the peripheral insulin sensitivity, owing to increased gut microbial diversity (Vrieze et al., 2012). Prediabetic and overweight Danish adults with insulin resistance and dyslipidemia showed perturbed gut microbiota with low abundance of *Akkermansia muciniphila* and the genus *Clostridium* (Allin et al., 2018; Harsch and Konturek, 2018). A metagenomic-wide association study suggested the association between type 2 diabetes and gut dysbiosis, a reduction in the butyrate-producing gut bacteria and an increase in the opportunistic pathogenic bacteria (Qin et al., 2012). Thus, a strong interaction between host genetics, gut microbiota and diet exists, thereby determining the metabolic phenotype. Furthermore, the metabolic phenotype can be manipulated by changing the microbiota composition as suggested by the fecal transplant experiments, establishing the causative role of microbiota in regulating the whole-body metabolism (Ussar et al., 2015; Boulangé et al., 2016; Muscogiuri et al., 2019).

The mechanism by which microbiota can affect the whole-body metabolism and the inflammation involves the microbial metabolites that reach the circulation and the vagus nerve, thereby affecting the host metabolism, inflammation, and neuroendocrine secretions (Cani, 2019; Scheithauer et al., 2020; Yang et al., 2021; Figure 2). The elevated level of microbially produced imidazole propionate was found in the portal and circulating plasma of subjects with type 2 diabetes compared to healthy control group (Koh et al., 2018). Furthermore, this study showed that imidazole propionate impairs insulin signaling through mTORC1 by inhibiting tyrosine phosphorylation of insulin receptor substrates, leading to its proteasomal degradation. Similarly, indole, another bacterial metabolite, reduces the LPS-induced upregulation of pro-inflammatory mediators in liver, and its oral administration before LPS injection reduces the expression of key inflammatory proteins (Beaumont et al., 2018). The gut microbiota composition has an important impact on the metabolism of ingested food and nutrients. Some metabolites may be linked with the disease pathogenesis such as cardiovascular and metabolic diseases (Brown and Hazen, 2018; Scheithauer et al., 2020). The gut microbiota-derived metabolite trimethylamine *N*-oxide (TMAO) has been regarded as a risk factor for cardiovascular disease pathogenesis by promoting atherosclerotic lesions. Dietary choline and/or L-carnitine are the main sources which are required to produce TMAO. The studies from GF mice indicate the role of dietary choline and TMAO produced from the gut microbiota in atherosclerosis and macrophage cholesterol accumulation (Wang et al., 2011). Furthermore, the susceptibility to atherosclerosis can be transmitted to otherwise healthy mice by transplanting TMAO-producing gut microbiota obtained by mice fed with a diet rich in choline (Gregory et al., 2015). Increasing evidence suggests a crosstalk between the eCB system and microbiota in atherosclerosis (Moludi et al., 2018). The bile acid composition can also affect the composition of microbiota or conversely, microbiota can affect the metabolism of bile acids. The altered metabolism of bile acids has been associated with IBD, metabolic disorders, and colorectal cancer (Nagengast et al., 1995; Mudaliar et al., 2013; Tiratterra et al., 2018). The SCFAs, produced by the microbiota *Bifidobacterium* and *Akkermansia muciniphila* through intestinal fermentation of dietary fibers, have been inversely related with the low-grade inflammation, insulin resistance and type 2 diabetes (Canfora et al., 2015; Pedret et al., 2019). SCFAs, such as butyrate and propionate, can reduce hepatic lipid accumulation, and increase glucose tolerance in the animal models of diet-induced obesity and type 2 diabetes (den Besten et al., 2015). Similarly, in humans, circulating but not fecal acetate, propionate and butyrate were positively associated with the fasting GLP-1 levels, whereas circulating SCFAs were negatively related with the whole body lipolysis, free fatty acids and triacylglycerols (Müller et al., 2019).

### Gut-Brain Axis Effects on the Regulation of Hypothalamic-Pituitary-Adrenal Axis

Numerous studies have confirmed the interaction between gut microbiota and hypothalamic-pituitary-adrenal (HPA) axis, suggesting that the gut microbiota composition might affect the stress-induced activation of the HPA axis (Figure 2). GF rats show increased levels of corticosterone (CORT) in the open field test compared to SPF rats and show increased expression of stress-related corticotropin releasing hormone (CRH) in the hypothalamus, while show reduced glucocorticoid receptor (GR) expression in the hippocampus (Crumeyrolle-Arias et al., 2014). Postnatal disruption of microbiota colonization can affect the neuroendocrine response and the connectivity of neuronal circuits regulating the stress response. The HPA axis responds differently to restrain stress in GF mice, SPF mice and gnotobiotic mice. The GF mice show increased plasma ACTH and CORT levels under restraint stress (Sudo et al., 2004). Importantly, the changes were not limited to the HPA axis, but the cortex and hippocampus showed reduced levels of BDNF expression compared to SPF mice, suggesting the effect of gut microbiota composition on the CNS. The most striking finding of this study was that the hyperresponsiveness of HPA axis can be corrected by administering *Bifidobacterium infantis* or transplanting microbiota from SPF mice only during the early stage but not at the later stage, suggesting the role of microbiota in the early programming of neuroendocrine response of the HPA axis (Sudo et al., 2004). Recently, using GF and SPF mice, it has been shown that the microbial colonization decreases the colonic expression of 11-β-hydroxysteroid dehydrogenase type 2 (11HSD2), CRH, corticotropin releasing hormone receptor 2 (CRHR2), urocortin 2 (UCN2) and its receptor during chronic psychosocial stress, but increases the expression of genes associated with the inflammatory cytokines, such as tumor necrosis factor-α (TNF-α), interferon-γ (IFN-γ) and interleukin-6 (IL-6) (Vodička et al., 2018). This study further confirmed that the absence of microbiota increases the expression of tyrosine hydroxylase (TH), PNMT, melanocortin receptor 2 (MC2R), steroidogenic acute regulatory protein (StAR) and cytochrome P450 family 11 subfamily A member 1 (CYP11a1) in the adrenal, while in the pituitary, it increases the expression of FKB prolyl isomerase 5 gene, which regulates GR sensitivity. In addition to neuronal effects mediated by the gut-brain axis, the metabolites released from the gut microbiota can directly modulate host physiology. Indole produced from the gut bacteria has recently been shown to affect the adrenal medulla in mice and modulate the expression of PNMT gene involved in the catecholamine biosynthetic pathway (Mir et al., 2020).

In rats, chronic prenatal stress leads to the hyperresponsiveness of the HPA axis to stress later in life and affects the development of the enteric nervous system with marked reduction in the colonic innervation density and augmented secretory response of colon after catecholaminergic stimulation (Golubeva et al., 2015). Furthermore, prenatal stress also alters the microbiota composition and reduces *Lactobacillus*, while increasing *Oscillibacter*, *Anaerotruncus*, and *Peptococcus.* In mice, the transfer of early prenatal stress-mediated disruption of maternal microbiota to offspring can affect the gut transcriptome and the hypothalamic gene expression during chronic stress later in adulthood (Jašarević et al., 2018). The HPA response, as measured by the plasma CORT levels in adult offspring 15 min after restraint stress, showed higher activity in vaginally delivered prenatally stressed males compared with the control male offspring. Furthermore, transfer of maternal vaginal microbiota from stressed mothers to unstressed pups delivered by cesarean section had similar effects on the stress-response to those observed with the prenatally stressed pups, suggesting the role of maternally transferred microbiota in stress response during adulthood (Jašarević et al., 2018). Similarly, stress during pregnancy also affects the microbiota composition of the mother, and the microbiota of both mother and offspring can affect the development and programming of the HPA axis, leading to aberrant HPA response and social behavior later in life (de Weerth, 2017; Gur et al., 2019; Frankiensztajn et al., 2020). Early life stress regulates the responsiveness of the HPA axis later in life, and therefore *in utero* exposure or neonatal stress can lead to dysbiosis and affect the development of the HPA axis. In human infants, it was shown that maternal prenatal stress, as confirmed by the elevated levels of maternal salivary cortisol, perturbs the microbiota composition with significantly increased abundance of *Proteobacterial* groups and decreased abundance of *Lactobacillus* and *Bifidobacterium*, leading to enhanced GI inflammation (Zijlmans et al., 2015). Similarly, stressed rats pups with limited nesting show increased basal CORT at weaning with decreased microbial diversity and increased gut permeability. This effect was more prominent in females than males, suggesting the influence of sex on microbiota induced changes in the HPA axis (Moussaoui et al., 2017). In addition to chronic stressors, acute stressors can also affect the colonic microbiota composition. In such experiment, an acute brief exposure to social stressor significantly alters the relative abundance of microbiota, decreasing the beneficial *Lactobacillus* genus (Galley et al., 2014a).

## Microbiota-Gut-Brain Axis and Stress Responses-Potential Role of the Microbiota-Gut-Brain Axis in Stress Resilience

Here, we will summarize those findings that relate the microbiota-gut-brain axis to stress-related disorders (Figure 2). In this regard, the first evidence comes from the fact that IBS shows high co-morbidity with stress-related disorders, such as anxiety and depression (Whitehead et al., 2002). Indeed, it has been proposed that modulation of the microbiota (e.g., with the use of probiotics) as a novel strategy not only for the treatment of IBS, but also as an adjuvant for psychiatric treatment of anxiety and depression.

Furthermore, the GF mice as animal model are instrumental to elucidate the impact of the microbiota in both health and pathological conditions. Thus, GF mice, in the absence of gut microbiota, showed increased HPA axis activity in response to stress (Sudo et al., 2004; Neufeld et al., 2011), suggesting an increased susceptibility to stress-related responses such as anxiety. In line with these findings, the absence of gut microbiota increases anxiety behavior in GF adult mice (Neufeld et al., 2011). Furthermore, the BALB/c mouse line showed an enhanced anxiety-like behavior in comparison to the NIH Swiss mouse line (Bercik et al., 2011a). Importantly, such behavioral phenotype was swapped by gavage administration of the cecal microbiota to GF mice from these two mouse lines, conferring the behavioral profile of the donor (Bercik et al., 2011a). Challenging this notion, some studies found that adult GF mice displayed reduced anxiety-like behavior in comparison to mice with gut microbiota (Diaz Heijtz et al., 2011; Neufeld et al., 2011). These data put forward the concept that intestinal microbiota influences anxiety-like behavior.

Moreover, using animal models of stress, it was described that stress can affect gut microbial composition (Gareau et al., 2007; Bharwani et al., 2016; Burokas et al., 2017; Pascual Cuadrado et al., 2021; Figure 2). Clinical studies also found changes in microbial composition in patients with a diagnosis of depression or anxiety disorder (Bastiaanssen et al., 2019). These findings suggest that stress induces a dysbiotic state with a decrease in commensal microbiota and an increase in opportunistic pathogens. The underlying mechanisms are complex, bidirectional, and still elusive. Stress might alter gut functions via activation of the sympathetic nervous system and/or the HPA axis by, for example, modifying gut permeability. Dexamethasone treatment, mimicking the stress response, increased GI permeability (Meddings and Swain, 2000). Changes in permeability increase mobility of LPS or other endotoxins from the lumen to the host and its interaction with the immune system, promoting the expression of inflammatory mediators in the circulation. Accordingly, changes in microbial composition by an antibiotic cocktail blunted the stressor-induced increase in circulating cytokines, such as IL-6, TNF-α, IFN-γ and MCP-1 (Bailey et al., 2011). Early life stress has been proposed as a model to study the gut-brain axis (O’Mahony et al., 2011). Rodents exposed to early life stress showed changes in gut functions, immune system, microbiota and an excessive stress response (O’Mahony et al., 2011). Thus, early life stress causes an inflammatory immune phenotype characterized by increased pro-inflammatory cytokines (IL-1β, IL-6, TNF-α), which are also associated with depression (Hodes et al., 2015; Dutcher et al., 2020). In maternal separated rats, probiotic interventions reversed stress-induced behavioral deficits as well as normalized blood cytokines levels (Desbonnet et al., 2010).

Furthermore, pre- and probiotic interventions have been used extensively to investigate the physiological functions of the microbiota-gut-brain axis. While probiotics are living microorganisms that exert beneficial effects on the health of the host after ingestion, prebiotics provide food favoring the growth of beneficial microbes. Several studies described the treatments using prebiotics, postbiotics or the combination of both can mitigate stress-induced anxiety- and depression-like behavior in animal models (Gareau et al., 2007; Bravo et al., 2011; Burokas et al., 2017; Marin et al., 2017), but also in human studies (Messaoudi et al., 2011). For example, chronic treatment (for 4 weeks) with probiotic *Lactobacillus rhamnosus (JB-1)* caused a decreased anxiety- and depressive-like behavior in healthy mice during elevated plus maze and forced swimming test, respectively, concomitant with a reduced acute stress-induced CORT levels (Bravo et al., 2011). *Lactobacillus rhamnosus* modified gene expression levels of the GABAergic system, the main inhibitory neurotransmitter system in the brain, in several key brain areas of the stress system (Bravo et al., 2011). Notably, the probiotic-related effects on neurochemical changes as well as in behavior were abolished in vagotomized mice (Bravo et al., 2011), suggesting an instrumental role of the vagus nerve in the beneficial responses mediated by the probiotics. In another study, probiotic treatment diminished the maternal separation stress-induced changes in gut physiology and normalized elevated CORT levels in rats (Gareau et al., 2007), in this case, indicating a crosstalk between the microbiota and the HPA axis. Additionally, a chronic combined prebiotic treatment protected against chronic social stress-induced anxiety-like behaviors, reduced social interaction, stress-induced elevetations in proinflammatory cytokines, and stress-induced alterations in the microbiome (Burokas et al., 2017). The beneficial effect of pre- and probiotic interventions on emotional responses seems to be more pronounced following stress. Accordingly, probiotic supplementation was effective in decreasing depressive-like behavior in stressed rats but not in non-stressed rats (Desbonnet et al., 2010). In healthy humans, probiotic supplementation for 30 days alleviated physiological distress (Messaoudi et al., 2011). In summary, these findings suggest that specific probiotic strains might be applied as treatment against mental health disorders.

It is well established that the eCB system strongly regulates stress response (Lutz et al., 2015; Morena et al., 2016). Regarding the specific role of the intestinal eCB system in the regulation of stress responses, only few studies have addressed this appealing hypothesis. In a fundamental study, Guida and coworkers examined the impact of antibiotic-induced dysbiosis on the intestinal eCB system as well as on depressive-like behaviors (Guida et al., 2018). In short, antibiotic-induced perturbation of the microbiota caused a depressive-like phenotype in the tail suspension and social interaction test. At the molecular level, the antibiotic-induced dysbiosis led to dramatic changes in the overall gut microbial composition, local inflammation, and decreased AEA levels in the duodenum (Guida et al., 2018). Importantly, some of these changes were reversed after probiotic administration (Guida et al., 2018). Concerning the stress-induced changes in gut motility, a peripherally restricted CB1R agonist was able to normalize the stress effects in motility, pointing out that intestinal CB1R is a key regulator of gut dysfunctions caused by stress (Keenan et al., 2015). In another crucial study, fecal microbiota transplantation of chronic mild stressed mice, a mouse model of depression, caused depressive-like behavior in the recipient mice (Chevalier et al., 2020). Notably, molecular and behavioral alterations in the recipient mice were linked to a reduced serum lipid precursors for the production of eCB ligands as well as decreased eCB levels in the brain (Chevalier et al., 2020), supporting the stress-induced “hypocannabinergic state” hypothesis (Bosch-Bouju et al., 2016; Bluett et al., 2017), where gut microbial composition might be instrumental. Indeed, alterations of the gut microbiota profoundly impact the intestinal eCB tone (Manca et al., 2020). Here, the authors showed that GF mice exhibited profound alterations in the intestinal eCB system, including a changes in gene expression of CB1R, GPR55 and PPARα in the intestine, in comparison to control mice, whereas fecal transplantation partially reversed these differences (Manca et al., 2020), strongly supporting that microbiota composition has a profound impact in the intestine eCB tone. Alterations of the intestinal eCB system may provide a link between stress and chronic abdominal pain (Rousseaux et al., 2007; Hong et al., 2015; Guida et al., 2020), a common feature of GI disorders, anxiety and depression. In a model of chronic stress-induced visceral pain, CB1R mRNA levels were downregulated in L6-S2 dorsal root ganglia (DRG) that innervate pelvic organs including the colon (Hong et al., 2015). Furthermore, low vitamin D dietary intake-induced allodynia was linked with lowered microbial diversity and reduced 2-AG levels in the colon (Guida et al., 2020). Interestingly, other studies have pointed out that the CB2R expression in the epithelial cells of the intestine might have an analgesic effect in visceral pain (Rousseaux et al., 2007).

Based on the above evidence, an attractive hypothesis points to the role of the gut microbiota as a potential resilience factor against stress-related pathologies. Indeed, the idea that diet might influence emotional responses has been established many years ago. Some studies found that certain species of bacteria are more abundant in resilient mice in comparison to control or susceptible mice (Marin et al., 2017; Yang et al., 2017; Bassett et al., 2019; Pascual Cuadrado et al., 2021). Recently, we have demonstrated differences in gut microbial composition associated with a resilience outcome to a single trauma in mice (Pascual Cuadrado et al., 2021). Interestingly, we found an increased abundance of *A. municiphila* in resilient mice as compared to susceptible littermates. In another study, oral administration of the probiotic *Bifidobacterium* increased the number of resilient mice to chronic social defeat paradigm (Yang et al., 2017). Conflicting results have also been reported and some studies have not found an association between microbiota composition and emotional responses (Tsilimigras et al., 2018).

Both clinical and animal studies described a prominent feature of sexual differences in the incidence of mental health disorders, with almost two-fold higher prevalence of anxiety and depression in women (Kessler, 2003; Goodwill et al., 2019). Importantly, accumulating evidences describes that alterations in female hormones during adolescence, pregnancy, postpartum or menopausia are associated with changes in microbiota composition, immune responses and increased vulnerability to mood disorders (Jašarević et al., 2016; Audet, 2019). Moreover, alterations in sex-specific microbiota composition may rely on gender sensitivity to environmental factors such as stress reactivity. The mechanistic insights of the sex hormone-related changes in microbial composition are not fully understood yet. In any case, sex differences in the microbiota-gut-brain axis function might contribute to the increased vulnerability of anxiety and depression in both women and female rodents. Nevertheless, it is still an unexplored research area, and more studies are needed to unravel the sexual dimorphism in the microbiota-gut-brain axis related to physiological and pathophysiological functions.

Overall, these findings underline the link between microbiota-gut-brain axis and stress responses and point out that underlying mechanisms are complex, intertwined, and bidirectional (Figure 2). Indeed, it might also be plausible that several of the described mechanisms are working in parallel. We also put forward the notion that certain gut microbial populations provide resilience to stress-related pathologies, where the intestinal eCB system might play a key role. Thus, those approaches that favor the growth of a “beneficial” bacteria could be favorable in conferring resilient mechanisms against traumatic or stressful events. Furthermore, the aforementioned evidence showed that the crosstalk between microbiota and the intestinal eCB might play a prominent role in the regulation of emotional responses. Further studies will have to elucidate whether it might serve a novel therapeutic target to confer resilience against stressful events.

## Changes in Gut Microbial Composition Associated With Pathological Conditions, Studies in Animal Models

Gut microbiota abundance and composition change during different physiological and pathophysiological conditions such as gut inflammation, obesity, diabetes, and stress (Table 1). In chemical-induced colitis models higher relative abundance of orders Enterobacteriales, Verrucomicrobiales, and Deferribacterales while lower abundance of Bacteroidales have been reported (Lupp et al., 2007; Berry et al., 2012). Notably, the relative abundance of the family *Bacteroidaceae* increases while *Rikenellaceae* decreases. The relative abundance of *Akkermansia* increases in dextran sodium sulfate-induced colitis model (Berry et al., 2012). Similarly, pathogen-induced gut inflammation is associated with increased abundance of Gammaproteobacteria. Mice that are genetically susceptible to gut inflammation, such as IL-10 deficient mice, harbor higher numbers of *Escherichia coli* than healthy controls (Wohlgemuth et al., 2009). Metabolic disorders are also associated with the changes in microbiota. In diabetic (db/db) mice, higher abundance of microbiota from phylum Firmicutes, Proteobacteria, and Fibrobacteres have been found (Geurts et al., 2011). More importantly, the genera *Prevotella, Odoribacter*, and *Rikenella* are found exclusively in db/db mice, whereas the relative abundance of *Tannerella* increases by several fold in db/db mice (Geurts et al., 2011). Similarly, genetically (ob/ob) and high fat diet-induced obese mice have lower abundance of Bacteroidetes with a proportional increase in Firmicutes (Ley et al., 2005; Cani et al., 2007). In high fat diet fed mice, lower abundance of *Bifidobacterial* and *Eubacterium rectale-Clostridium coccoides group* compared to controls have been reported (Ley et al., 2005). Thus, obesity and metabolic syndrome are closely associated with changes in the diversity of gut microbiota. Similarly, stress exposure can cause gut dysbiosis. In rodents, restraint stress increases relative abundance of gut microbiota belonging to phylum Firmicutes and Deferribacteres while reduces Actinobacteria (Galley et al., 2017). In addition, social stress caused by exposure of aggressor mice affects the relative abundance of microbiota (Gautam et al., 2018). Particularly, the phylum Firmicutes and Bacteroidetes are more vulnerable to this kind of stress. The proportion of Firmicutes and Verrucomicrobia decrease after exposure to the aggressor. The social stress in rodents produce the most striking changes in the relative abundance of microbiota in the genus *Lactobacillus*, *Akkermansia*, *Oscillospira, Coriobacteriaceae*, and *Anaeroplasma* (Bharwani et al., 2016; Gautam et al., 2018). In a PTSD mouse model, we recently identified an increased abundance of *Akkermansia muciniphila* in resilient mice in the analysis of different taxa inhabiting the gut (Pascual Cuadrado et al., 2021). The prenatal stress and the disruption of mother-infant bond lead to stress related behavioral disorders as well as changes in the gut microbiota. Adult rats exposed to maternal separation show behavioral deficits, which can be reversed by the chronic treatment with Bifidobacteria or by anti-depressive citalopram treatment (Desbonnet et al., 2010). Furthermore, a significant reduction in *Lactobacillus* species has also been observed in maternally separated pups (Gareau et al., 2007). Notably, the decreased numbers of bacteria in the *Lactobacillus* genus persist in the adulthood (at 4 months of age) in prenatal stress rats (Golubeva et al., 2015). In infant rhesus monkeys, the reduction in fecal *Lactobacilli* is associated with stress-related behavioral deficit (Bailey and Coe, 1999). The maternal gut microbiome undergoes temporal changes during pregnancy owing to drastic changes in the hormonal and metabolic profile to meet the nutrient and energy demand (Jašarević et al., 2017). Stress during early pregnancy increases the relative abundance of *Rikenellaceae*, *Odoribacter*, and *Mucispirillum* while decreases *Bacteroides* (Jašarević et al., 2017). On the other hand, stress during late pregnancy decreases Prevotella and S24-7 of phylum Bacterioidetes while increases *Ruminococcaceae* of phylum Firmicutes (Jašarević et al., 2017).

**TABLE 1:** Changes in the microbiota composition associated with different pathological conditions in animal models.

Animal models	Bacterial abundance	References
**Gut inflammation**		
Dextran sodium sulfate (DSS)-induced colitis model	Decrease in phylum Bacteroidetes Increase in order Clostridiales, Verrucomicrobiales, and Anaeroplasmatales Decrease in order Bacteroidales Increase in family *Ruminococcaceae, Bacteroidaceae, Enterobacteriaceae, Deferribacteraceae*, and *Verrucomicrobiaceae* Increase in bacteria *Enterococcus faecalis*	Lupp et al., 2007; Berry et al., 2012
Citrobacter rodentium (CR) infection mediated inflammation model	Decrease in phylum Bacteroidetes Increase in phylum Firmicutes, and Proteobacteria Decrease in order Bacteroidales Increase in order Enterobacteriales and Bacillales Increase in family *Enterobacteriaceae*	Lupp et al., 2007
IL-10 deficient mouse	Increase in class Gammaproteobacteria Decrease in order Bacteroidales Increase in bacteria *Escherichia Coli*	Lupp et al., 2007; Wohlgemuth et al., 2009
A20^IEC/myel–KO^ mouse	Increase in phylum Deferribacteres Increase in bacteria *Mucispirillum schaedleri*	Vereecke et al., 2014
**Metabolic disorder**		
db/db mouse	Increase in phylum Proteobacteria Increase in class Deltaproteobacteria Increase in genus *Odoribacter, Prevotella, Rikenella, Tannerella*, and *Barnesiella*	Geurts et al., 2011
ob/ob mouse	Decrease in phylum Bacteroidetes Increase in phylum Firmicutes Increase in class Mollicutes	Ley et al., 2005; Turnbaugh et al., 2006
HFD-induced obesity model	Decrease in phylum Bacteroides Increase in phylum Firmicutes Increase in class Erysipelotrichi and Bacilli Increase in family *Rikenellaceae* and *Lachnospiraceae* Decrease in phylum Bacteroidetes Decrease in genus *Prevotella*, *Lactobacillus* and *Bifidobacterium* Decrease in bateria *Eubacterium rectale*, and *Clostridium coccoides*	Cani et al., 2007, 2008; Turnbaugh et al., 2008; Fleissner et al., 2010; Daniel et al., 2014; Pyndt Jørgensen et al., 2014
**Stress models**		
Maternal separation stress	Decrease in genus *Lactobacilllus*	Bailey and Coe, 1999; Gareau et al., 2007; Desbonnet et al., 2010
Prenatal stress	Decrease in phylum Actinomycetota and Bacteroidetes Increase in genus *Oscillibacter, Anaerotruncus, Peptococcus, Odoribacter, Mucispirillum*, and *Escherichia* Decrease in genus *Lactobacillus* Decrease in family *Streptococcaceae* Increase in family *Rikenellaceae*	Golubeva et al., 2015; Jašarević et al., 2017
Restraint stress	Increase in phylum Firmicutes and Actinomycetota Decrease in family Porphyromonadaceae, and *Lactobacillaceae* Increase in family *Ruminococcaceae* and *Lachnospiraceae* Decrease in genus *Tannerella*, *Lactobacillus, Adlercreutzia, and Sarcina* (in females) Increase in genus *Oscillospira*, and *Bifidobacterium* Increased colonization by *Citrobacter rodentium, and Ruminococcus* gnavus in females but decreased in males	Bailey et al., 2010; Galley et al., 2014b,^2017^; Tsilimigras et al., 2018
Social stress model	Decrease in genus *Bacteroides* Increase in genus *Clostridium* and *Ponticaulis* Decrease in family *Lactobacillaceae* and *Porphyromonadaceae* Decrease in genus *Lactobacillus, Parabacteroides, Akkermansia* Decrease in unclassified *Firmicutes*, and unclassified *Bacilli* Decrease in genus *Bifidobacterium* in susceptible mice *Decrease in Lactobacillus reuteri*	Bailey et al., 2011; Galley et al., 2014a; Yang et al., 2017; Gautam et al., 2018
Unpredictable chronic mild stress	Increase in family *Ruminococcaceae* and *Porphyromonadaceae* Decrease in family *Lactobacillaceae*	Chevalier et al., 2020
Single-trauma model	Increase in genus *Bacteroides* in resilient mice Decrease in phylum Firmicutes in resilient mice Increase in bacteria *Akkermansia muciniphila, Bacteroides acidifacians* and *Clostridium citroniae* in resilient mice	Pascual Cuadrado et al., 2021

*Changes in comparison to the control group.*

## Potential Interventions Targeting the Endocannabinoid System in Stress-Related Disorders

As outlined above, there is a mutual interaction between gut eCB signaling and microbiome, which thereupon can impact the organism’s stress reaction via the gut-brain axis and may contribute to a protection from stress-related disorders. Hereby, several mechanisms may be involved in, including the regulation of intestinal barrier integrity, influences on immune modulation and on the enteroendocrine system, and mediators from the microbiome passing into the body. The role of the eCB system is particularly apparent in pathological states such as in rodent models of obesity and type-2 diabetes, and gut inflammation (e.g., IBD-like pathologies, but not under normal physiological conditions. This is consistent with the fact that the eCB system acts to enable the homeostatic state of the organism and gets into action under internal and external challenges (Di Marzo, 2009). Chronic treatment of obese rodents with THC led to altered microbiota with an increased Firmicutes:Bacteroidetes ratio, and concomitantly to the reduction of the obese state (Cluny et al., 2015). The mechanistic underpinnings are not understood, and the open issue remains whether the action of THC on body’s cannabinoid receptors (e.g., through desensitization of the CB1R) reversed the obese state, which in turn altered the microbiota composition. Chronic treatment with capsaicin, a TRPV1 agonist, in obese rodents increased the levels of butyrate-producing *Ruminococcaceae* and *Lachnospiraceae*, while leading to decreased levels of LPS production. Fecal microbiota transplantation in GF mice showed that capsaicin-induced protection against HFD-induced obesity was transferrable, indicating the crucial role of the microbiota (Kang et al., 2017). Using the obese diabetic ob/ob mouse model, capsaicin treatment revealed beneficial effects on glucose homeostasis, with significantly increased Firmicutes/Bacteroidetes ratio at the phylum level as well as increased *Roseburia* abundance and decreased *Bacteroides* and *Parabacteroides* abundances at the genus level (Song et al., 2017). Similarly, in another study, such a treatment was shown to improve glucose homeostasis via GLP-1, and concomitantly, microbiota composition was changed, with increased abundance of *Bacteroides* genera. Microbiota transplantation experiments revealed that the beneficial effect of capsaicin was transferable (Hui et al., 2020). CBD has drawn quite some attention due to its various beneficial effects, e.g., regarding anxiety and inflammation. In a recent study, the treatment of mice with CBD-enriched cannabis extracts was investigated on the gut microbiome and associated histomorphological and molecular changes in the mouse gut mucosa (Skinner et al., 2020). Increases in the relative abundance of the probiotic *Akkermansia muciniphila* was detected. In contrary, however, increases of pro-inflammatory cytokines and chemokines (including Il1ß, Cxcl1, and Cxcl2) were observed in the colon tissue, and decreased expression of Muc2 (a gene associated with gut integrity). These observations raise the question on the long-term effects of therapeutic CBD application in humans on the microbiome. Of note, the mice used were not in a pathological state, thus, leaving the issue that the situation might be different if there were an induced pathological state, such as obesity or gut inflammation. In fact, in a mouse model of colitis, fish oil (20 mg/mouse) and CBD (1 mg/kg) were ineffective when given alone, but when co-administered reduced all inflammatory markers and the increased intestinal permeability, but not the behavioral impairments (anxiety, object recognition) (Silvestri et al., 2020). *Akkermansia muciniphila*, a species suggested to mediate anti-inflammatory action in colitis, was increased by the experimental colitis after 12 days, but its levels were significantly elevated by the combined treatment already at day 8, indicating that the treatment intervention enhanced the elevation of *Akkermansia muciniphila* upon induction of experimental colitis, possibly thereby enabling the beneficial effects of these species. These data reveal the potential of phytocannabinoids in the modulation of the microbiome, and consequently in gut functions, immune status and finally to behavioral changes. Yet, regarding the modulation of stress-related disorders, the evidence is sparse and needs specific investigations, but considering the link between stress and dysregulations of the microbiome and gut functions, such experiments are promising and will shed light onto new aspects of the action of phytocannabinoids.

### Future Prospective

Studies done so far confirm the role of the eCBs and related compounds, CB1R and CB2R in GI disorders and gut-brain axis. However, the role of other components related to the eCB system, such as GPR40, GPR41, GPR43, GPR55, GPR119, GPR120, and TRPV1-4 and PPAR-γ, is still elusive. Interestingly, some of these GPCRs are activated by intestinal lipid-signaling molecules affecting the release of GLP-1 and GIP. GPR40, GPR119, and GPR120 have been identified in K- and L-cells of the intestine (Reimann et al., 2008; Parker et al., 2009). Therefore, further research to unravel the roles of enteroendocrine cells in lipid sensing is required. This is crucial especially when the role of the vagus nerve and gut-brain axis has already been suggested in fat intake. Most of these receptors are also targets of phytocannabinoids. For further complication, CBD is also an inverse agonist of three other orphan GPCRs, namely GPR3, GPR6, and GPR12. Therefore, further research should shed light on their expression and functional role in the GI tract as well as their interaction with the gut-brain axis. Future studies to investigate the roles of these receptors are warranted to exploit their therapeutic potential in GI and neurological disorder.

## Author Contributions

RS, BL, and IRA wrote the manuscript. All authors contributed to the article and approved the submitted version.

## Conflict of Interest

The authors declare that the research was conducted in the absence of any commercial or financial relationships that could be construed as a potential conflict of interest.

## Publisher’s Note

All claims expressed in this article are solely those of the authors and do not necessarily represent those of their affiliated organizations, or those of the publisher, the editors and the reviewers. Any product that may be evaluated in this article, or claim that may be made by its manufacturer, is not guaranteed or endorsed by the publisher.
